# A new mode of DNA binding distinguishes Capicua from other HMG-box factors and explains its mutation patterns in cancer

**DOI:** 10.1371/journal.pgen.1006622

**Published:** 2017-03-09

**Authors:** Marta Forés, Lucía Simón-Carrasco, Leiore Ajuria, Núria Samper, Sergio González-Crespo, Matthias Drosten, Mariano Barbacid, Gerardo Jiménez

**Affiliations:** 1 Institut de Biologia Molecular de Barcelona-CSIC, Barcelona, Spain; 2 Molecular Oncology Programme, Centro Nacional de Investigaciones Oncológicas, Madrid, Spain; 3 ICREA, Barcelona, Spain; University of Michigan, UNITED STATES

## Abstract

HMG-box proteins, including Sox/SRY (Sox) and TCF/LEF1 (TCF) family members, bind DNA via their HMG-box. This binding, however, is relatively weak and both Sox and TCF factors employ distinct mechanisms for enhancing their affinity and specificity for DNA. Here we report that Capicua (CIC), an HMG-box transcriptional repressor involved in Ras/MAPK signaling and cancer progression, employs an additional distinct mode of DNA binding that enables selective recognition of its targets. We find that, contrary to previous assumptions, the HMG-box of CIC does not bind DNA alone but instead requires a distant motif (referred to as C1) present at the C-terminus of all CIC proteins. The HMG-box and C1 domains are both necessary for binding specific TGAATGAA-like sites, do not function via dimerization, and are active in the absence of cofactors, suggesting that they form a bipartite structure for sequence-specific binding to DNA. We demonstrate that this binding mechanism operates throughout *Drosophila* development and in human cells, ensuring specific regulation of multiple CIC targets. It thus appears that HMG-box proteins generally depend on auxiliary DNA binding mechanisms for regulating their appropriate genomic targets, but that each sub-family has evolved unique strategies for this purpose. Finally, the key role of C1 in DNA binding also explains the fact that this domain is a hotspot for inactivating mutations in oligodendroglioma and other tumors, while being preserved in oncogenic CIC-DUX4 fusion chimeras associated to Ewing-like sarcomas.

## Author summary

Transcription factors bind specific sites in the genome via discrete protein domains that recognize their target DNA sequences. One such domain is the HMG-box, which is found in many chromatin and transcriptional regulators across species. Two salient groups of HMG-box proteins are the Sox/SRY and TCF/LEF1 factors, which are involved in multiple developmental and signaling processes. Extensive genetic and molecular studies have shown, however, that both groups of proteins do not simply bind DNA through their HMG-box, but rely either on additional protein domains or associated factors for targeting their correct sites in the genome. In this work, we have focused on another HMG-box protein, Capicua (CIC), which has recently emerged as an important mediator of Ras/MAPK signaling in both *Drosophila* and mammals. We find that the HMG-box of CIC does not bind DNA alone and instead requires a separate conserved motif (C1) present in all CIC proteins. The C1 domain is restricted to CIC proteins and exhibits several properties that distinguish it from Sox and TCF domains involved in DNA binding. Thus, CIC proteins represent a separate sub-family of HMG-box factors that have evolved an independent mechanism for enhancing the DNA-binding capabilities of their HMG-box. Notably, our results also explain distinct patterns of human *CIC* mutations that either inactivate CIC tumor suppressor function or produce oncogenic fusions between CIC and the DUX4 activator factor.

## Introduction

HMG-box factors are abundant nuclear proteins with highly diverse functions in the cell. They contain one or more HMG-box domains that bind the minor groove of DNA, bending the duplex away from the interaction site. Proteins with tandem HMG-box domains usually function as architectural and chromatin factors and do not exhibit DNA sequence specificity. In contrast, proteins with a single HMG-box domain, including Sox/SRY (Sox) and TCF/LEF1 (TCF) transcription factors, function as developmental regulators and bind specific AT-rich motifs in enhancers and promoters (reviewed in refs. [1–3]). In most cases, however, this binding is not sufficient for appropriate target selection. For example, Sox proteins rarely act on their own and are often assisted by partner factors that bind next to the Sox sites, thereby stabilizing the complex and providing the specificity needed for in vivo function [3]. Once tethered to DNA, HMG-box proteins can exert their transcriptional effects through additional interactions with co-activators or co-repressors.

**Fig 1:**
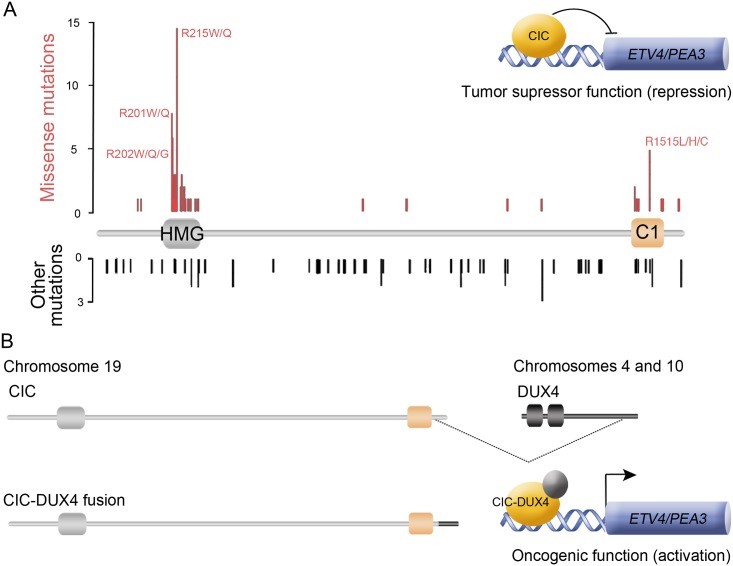
Patterns of *CIC* mutations in human OD and *CIC-DUX4* sarcomas. (A) Diagram of the CIC protein showing a set of curated mutations from the COSMIC database (http://cancer.sanger.ac.uk/cosmic). Only mutations corresponding to gliomas are shown. The tumor suppressor role of CIC in OD is thought to involve the repression of CIC targets such as the *ETV/PEA3* family genes [29, 30]. Note that missense mutations tend to cluster in the HMG-box and C1 domains. In contrast, nonsense and frameshift mutations (indicated as ‘Other mutations’) are distributed along the entire length of the protein, which is also consistent with a requirement for an intact C-terminal region. (B) Structure and function of oncogenic CIC-DUX4 fusions, which usually include most of the CIC protein (including the C1 domain) coupled to the C-terminal trans-activation domain of DUX4 [31,66]. The double homeodomain region of DUX4 is indicated by boxes.

The HMG-box protein Capicua (CIC) is a highly conserved transcriptional repressor distantly related to Sox and TCF factors [4]. Studies in *Drosophila* and mammals have shown that CIC controls multiple developmental decisions acting downstream of Receptor Tyrosine Kinase (RTK) signaling. In general, CIC represses RTK-responsive genes by binding to octameric TGAATGAA-like motifs in their promoters and enhancers, and this repression is relieved upon RTK-induced downregulation of CIC. In *Drosophila*, this mechanism controls anteroposterior and dorsoventral body patterning, intestinal stem cell proliferation, wing development, and other processes, providing a direct link between RTK activation and transcriptional derepression of CIC targets [5–14]. In mammals, CIC is similarly regulated by RTK signaling and controls essential processes such lung alveolarization and liver homeostasis [15–18]. Moreover, CIC has been implicated in distinct human pathologies including spinocerebellar ataxia type 1 [16,19] and various forms of cancer, particularly oligodendroglioma (OD) [20–23]. In cancer, CIC behaves mainly as a tumor and metastasis suppressor that is inactivated by somatic mutations [22–30], but it can also exert oncogenic effects resulting in Ewing-like sarcomas [31] (Fig 1). This latter role originates from chromosomal translocations where CIC becomes fused to a fragment of the DUX4 transcription factor [31–36]. CIC-DUX4 chimeras contain a nearly complete CIC sequence followed by the C-terminal portion of DUX4, which converts CIC into an activator and causes upregulation of CIC targets such as *ETV/PEA3* family genes [15,17,31].

Nevertheless, the mechanisms underlying CIC activity in normal and pathological processes are not well understood. One unresolved question concerns the role of a conserved domain present at the C-terminus of all CIC proteins. This domain (referred to as C1) does not resemble other known domains and appears to be functionally important. Thus, transgenic assays indicate that C1 is required for CIC repressor activity in early *Drosophila* embryos [9]. Also, systematic sequencing of human tumors has revealed multiple missense mutations mapping to the C1 sequence, arguing that C1 is essential for CIC function in suppressing growth and metastasis [22–27,30]. However, how C1 contributes to CIC function remains unknown.

In this work, we set out to investigate the mechanism of C1 action. Unexpectedly, we find that C1 plays a conserved essential role in CIC DNA binding activity. We show that neither the HMG-box nor the C1 domain is capable of binding to DNA separately, but instead function together to mediate efficient DNA binding in both *Drosophila* and human cells. Thus, CIC employs a new mode of DNA binding that distinguishes it from Sox and TCF proteins, which lack the C1 domain and employ other mechanisms for enhancing their target specificity. Furthermore, our results explain the distinct patterns of human *CIC* mutations in OD and Ewing-like sarcomas, since the C1 domain should be required for the DNA binding activities of both CIC and CIC-DUX4 in those pathologies, respectively.

## Results

### The C1 domain is essential for multiple developmental functions in *Drosophila*

**Fig 2:**
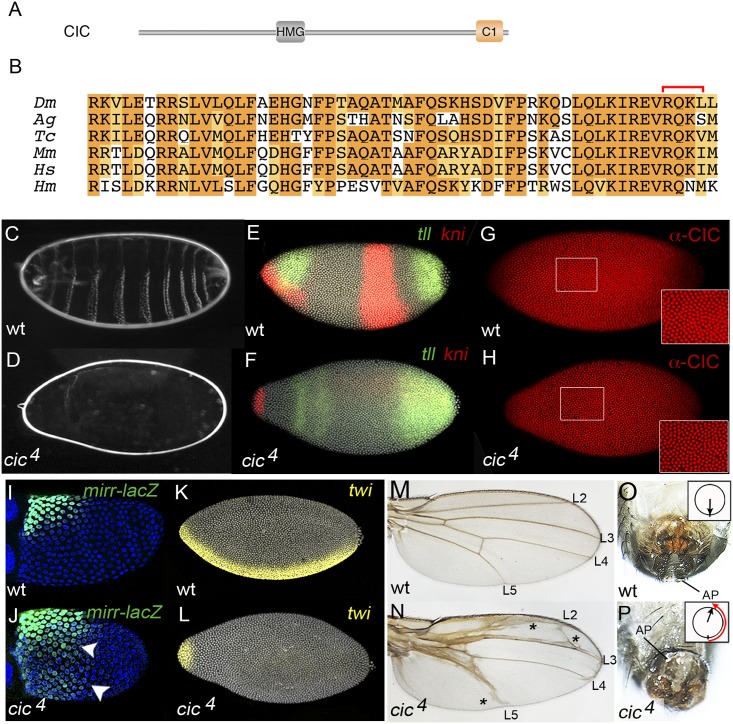
The C1 domain is required for multiple CIC functions in *Drosophila*. (A) Diagram of *Drosophila* CIC protein indicating the positions of the HMG-box and C1 domains. (B) Alignment of C1 domain sequences from *Drosophila* (*Dm*), *Anopheles* (*Ag*), *Tribolium* (*Tc*), mouse (*Mm*), human (*Hs*) and hydra (*Hm*). Light shading indicates similar residues. The four residues deleted by the *cic*^*4*^ mutation are indicated by a red bracket (see also S1 Fig.). (C, D) Cuticle of embryos derived from wild-type (C) and homozygous *cic*^*4*^ (D) females. The lack of patterning elements in the mutant reflects both suppression of trunk and abdominal regions as well as complete dorsalization of the embryo (see panels F and L). (E, F) Patterns of *tll* (green) and *kni* (red) mRNA expression in wild-type (E) and *cic*^*4*^ (F) embryos; nuclei are labeled with DAPI (grey). The mutant embryo shows expanded *tll* expression, which then causes repression of the abdominal *kni* domain. (G, H) Immunodetection of CIC protein in embryos from wild-type (G) and *cic*^*4*^ (H) females using anti-CIC antibody. Both backgrounds show similar levels of CIC nuclear accumulation (insets), indicating that the CIC^4^ mutant is stable but functionally inactive. (I, J) Patterns of *mirr-lacZ* reporter expression (green) in wild-type (I) and *cic*^*4*^ (J) stage-10 egg chambers; nuclei are labeled with DAPI (blue). Note the ventrally expanded expression of *mirr-lacZ* (arrowheads). (K, L) Expression of *twi* mRNA (yellow) in wild-type (K) and *cic*^*4*^ (L) embryos; nuclei are labeled with DAPI (grey). *twi* expression is severely reduced in the mutant embryo. Panels E, G, I, J and K are oriented with anterior to the left, dorsal up. (M, N) Wings from wild-type (M) and *cic*^*4*^ (N) adult flies; veins L2-L5 are indicated. The mutant displays thickened veins and ectopic vein material (asterisks). (O, P) External genitalia from wild-type (O) and *cic*^*4*^ (P) males; AP, anal plates. The *cic*^*4*^ individual exhibits a genital rotation phenotype (arrows indicate the genital arch-to-AP orientation).

The C1 domain is highly conserved in all CIC orthologs across metazoans. The conservation spans 40–45 amino acids with a highly invariable core of 11 residues at the C-terminal end (Fig 2A and 2B). Since CIC contains several conserved motifs that exert context-dependent functions [37], we tested the requirements of C1 in different *Drosophila* tissues. To this end, we used CRISPR-Cas9 to generate new *cic* alleles specifically disrupting the C1-coding sequence (S1 Fig). Among the isolated mutants, we selected an allele (designated *cic*^*4*^) that removes four amino acids in the resulting protein, including three highly conserved residues in the C1 core (Fig 2B).

*cic*^*4*^ homozygous flies are semilethal and show a range of developmental defects. During early embryogenesis, maternal CIC protein normally establishes the presumptive trunk and abdominal regions of the embryo by restricting *tailless* (*tll*) and *huckebein* (*hkb*) expression to the embryonic poles. At the poles, CIC is downregulated by Torso RTK signaling, thereby enabling localized induction of *tll* and *hkb* by broadly distributed activators [5,9,11,12] (reviewed in 4). In *cic* mutant embryos, *tll* expands towards the center of the embryo, which then causes repression of central patterning genes such as *knirps* (*kni*) and loss of central body regions. Consistent with a loss of maternal CIC function, *cic*^*4*^ females are fully sterile and lay embryos that lack all central thoracic and abdominal segmented regions (Fig 2C and 2D). Indeed, such embryos show clear derepression of *tll* and loss of the central *kni* stripe at the blastoderm stage (Fig 2E and 2F), indicating a failure of CIC-mediated repression. This effect is not caused by reduced CIC protein expression or stability, since *cic*^*4*^ embryos exhibit normal levels of CIC accumulation in blastoderm nuclei (Fig 2G and 2H), implying that the CIC^4^ mutant is functionally defective. A comparison with other *cic* mutations indicates that *cic*^*4*^ represents a strong hypomorphic allele (S2 Fig).

Next, we assayed the effects of *cic*^*4*^ in the follicular epithelium of the ovary. In this tissue, CIC organizes the future dorsoventral (DV) axis of the embryo by repressing *mirror* (*mirr*), thereby restricting its expression to dorsal positions. In *cic* mutant backgrounds, *mirr* becomes derepressed towards ventral regions and this leads to inappropriate repression of *pipe*, a gene that is critical for induction of ventral embryonic fates [6,8,38–40]. Consequently, the resulting progeny show a strongly dorsalized phenotype and loss of ventral patterning markers. We find that ovaries from *cic*^*4*^ females show derepressed *mirr* expression that is similar to that seen in strong *cic* mutant conditions (Fig 2I and 2J) [6,8]. In addition, embryos laid by *cic*^*4*^ females lack expression of *twist* (*twi*), a target of the maternal DV system that is normally activated in ventral positions (Fig 2K and 2L). Thus, C1 is also required for CIC repressor activity in the follicle cells.

Also, *cic*^*4*^ flies consistently show abnormal wings with extra vein tissue (Fig 2M and 2N). This phenotype reflects insufficient CIC activity during wing development, where CIC represses vein-promoting genes downstream of EGFR signaling [7,12] (see also below).

Finally, *cic*^*4*^ males are sterile and exhibit a severe genitalia rotation phenotype present in other strong *cic* mutants backgrounds [7] (Fig 2O and 2P). Thus, although we have not examined the requirement of C1 for all CIC functions in *Drosophila*, our results suggest that C1 mediates a key general aspect of CIC activity in this organism.

### Replacing the HMG-box of CIC by a heterologous DNA binding domain renders C1 dispensable for repression

**Fig 3:**
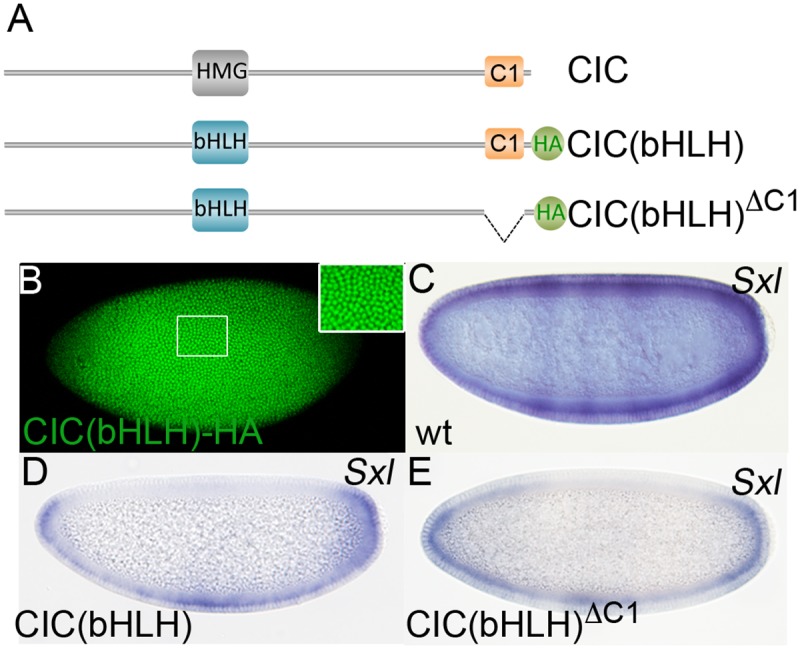
Fusion of CIC to a heterologous DNA binding domain bypasses the requirement for C1. (A) Structure of *Drosophila* CIC and CIC derivatives in which the HMG-box has been replaced by the bHLH domain of Hairy. The CIC(bHLH) and CIC(bHLH)^ΔC1^ proteins are tagged with the HA epitope and are thus discernable from endogenous CIC. (B) Expression of CIC(bHLH)-HA in embryos stained with anti-HA antibody. The inset shows a higher magnification view of nuclear CIC(bHLH)-HA accumulation. (C-E) *Sxl* mRNA expression in female wild-type (C) and transgenic embryos expressing CIC(bHLH) (D) and CIC(bHLH)^ΔC1^ (E). *Sxl* appears clearly repressed in both transgenic embryos.

Since C1 is important for CIC repressor function, we initially hypothesized that C1 might function as a repressor module that interacts with co-repressor factors. As a first test of this idea, we reasoned that replacing the HMG-box region of CIC with a heterologous DNA binding domain should produce a chimeric protein capable of repressing transcription in a C1-dependent manner. For this, we adopted an assay involving the basic-helix-loop-helix (bHLH) region of Hairy and the *Sex-lethal* (*Sxl*) gene [41,42]. We made a *cic* expression construct in which the HMG-box-coding region was replaced by the bHLH-coding region of Hairy and expressed this chimera, CIC(bHLH) (Fig 3A), in transgenic embryos under the control of *cic* genomic sequences. The CIC(bHLH) product was clearly detectable in nuclei of central and subterminal regions of the embryo (Fig 3B), whereas a CIC derivative lacking the HMG-box is mainly cytoplasmic [9], implying that the bHLH region targets CIC to the nucleus. One target recognized by the bHLH domain of Hairy is *Sxl*, a sex-determining gene activated exclusively in female embryos (Fig 3C). Hairy does not normally regulate *Sxl*, but it can do so when expressed earlier than usual (i.e. before or at early stage 5), by mimicking the activity of the Hairy-family repressor Deadpan [41,43]. Thus, premature Hairy expression causes inappropriate repression of *Sxl* and leads to female lethality. Accordingly, we find that early CIC(bHLH) expression driven by the maternal *cic* promoter causes extensive repression of *Sxl* except in polar regions of the embryo, where CIC(bHLH) nuclear levels and activity are lower in response to Torso signaling (Fig 3D). Consistent with this repression effect, CIC(bHLH)-expressing females show a clear ‘daugtherless’ phenotype as >95% of their progeny are males.

We then tested a CIC(bHLH) derivative lacking the C1 domain (Fig 3A, Materials and methods). Surprisingly, this construct behaves similarly to intact CIC(bHLH), causing evident repression of *Sxl* and lethality of the female progeny (Fig 3E). Thus, targeting CIC to the *Sxl* regulatory sequences via the Hairy bHLH domain renders CIC-mediated repression independent of C1. In concordance, we recently found that fusing C1 directly to a bHLH-containing fragment of Hairy does not lead to repression in the *Sxl* assay [37], whereas similar fusions with well-characterized repressor domains do [42,44–46]. Thus, although we cannot rule out that C1 could have an intrinsic repressor activity in other contexts, the fact that C1 is required for CIC but not CIC(bHLH) function points to a role of C1 in HMG-box-mediated DNA binding.

### The HMG-box and C1 domains are both needed for transcriptional repression and promoter targeting in human cells

**Fig 4:**
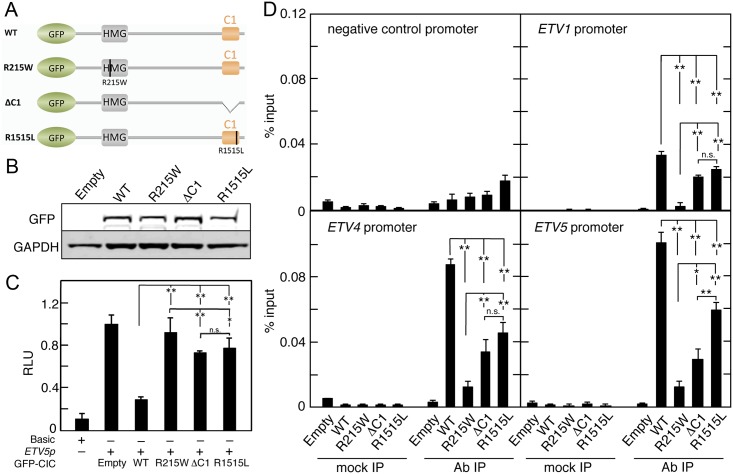
The C1 domain mediates CIC repression and promoter binding in human cells. (A) Diagram of GFP-tagged human CIC protein constructs tested in reporter and ChIP assays. Mutations in the HMG-box and C1 domains are indicated by vertical lines in both domains. (B) Western blot analysis of wild-type and mutant GFP-CIC fusion proteins stably expressed in Flp-In T-REx 293 cells using antibodies directed against GFP. GAPDH expression served as a loading control. (C) Relative luciferase expression levels driven by a promoter-less vector (*Basic*) or a synthetic promoter carrying CIC binding sites derived from the *ERM/ETV5* promoter (*ETV5p*), in the absence or presence of wild-type (WT) or the indicated mutant GFP-Cic constructs transfected into 293T cells. Luciferase values are expressed relative to the activity of the reporter co-transfected with empty *pcDNA5/FRT/TO* vector (Materials and methods). (D) ChIP assay using GFP antibodies in Flp-In T-REx 293 cells stably expressing wild-type (WT) or the indicated mutant GFP-CIC fusion proteins. Flp-In T-REx 293 cells stably transfected with an empty vector were used as a control (Empty). Association with the CIC binding elements in the *ETV1*, *ETV4* and *ETV5* promoters was analyzed by quantitative real-time PCR and normalized to the amount of input DNA. Statistical analysis was performed with one-way ANOVA followed by Tukey’s *post hoc* test; (*P<0.05 and **P<0.01); n.s., non significant.

Next, we tested the function of C1 in human cultured cells. To this end, we made a series of GFP-tagged human CIC derivatives carrying mutations in the HMG-box and C1 domains (Fig 4A). These constructs had similar levels of expression and were all detected in the nucleus, implying that tagging and mutagenesis did not differentially affect their stability or subcellular localization (Fig 4B; S3 Fig). Then, we assayed the repressor activities of the different constructs using a luciferase reporter under the control of a synthetic promoter carrying CIC binding sites (CBSs) derived from the *ETV5* promoter [15,17,31] (see Fig 4 legend). This reporter, *ETV5p*, was significantly repressed upon cotransfection of GFP-CIC (WT), whereas GFP-CIC constructs carrying either recurrent OD mutations affecting the HMG-box (R215W) or C1 (R1515L) domains [22,26,30], or a complete C1 deletion (ΔC1), showed reduced repressor activities relative to the intact control (Fig 4C). This indicates that the HMG-box and C1 domains are both required for CIC repressor activity in mammalian cells.

We then tested association of the above CIC mutants to endogenous *ETV/PEA3* gene promoters by chromatin immunoprecipitation (ChIP). As expected, the R215W mutation caused a reduction in ChIP signals relative to the control CIC protein (Fig 4D). Notably, both C1 mutations also diminished CIC promoter occupancy at all three *ETV/PEA3* gene analyzed. The reduction was more pronounced for the full C1 deletion, but the effect of the R1515L mutation was also clearly significant. These results indicate a requirement of both the HMG-box and C1 domains for binding of CIC to its target genes.

### The C1 domain cooperates with the HMG-box in DNA binding

**Fig 5:**
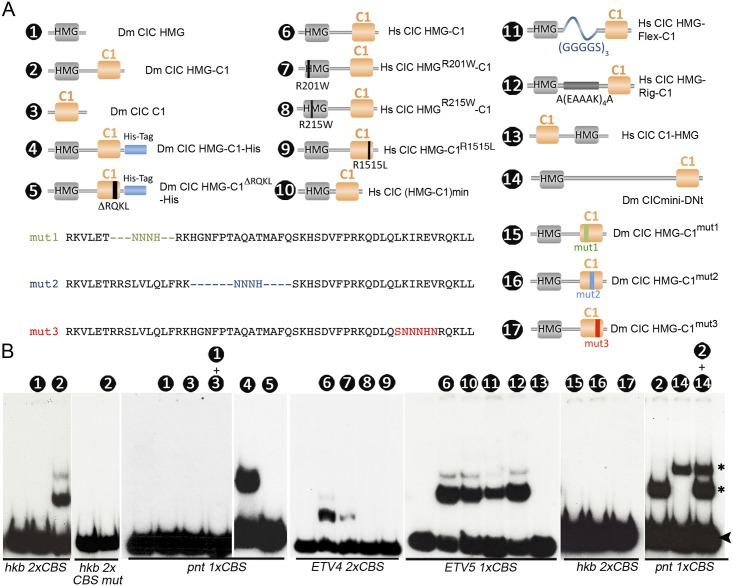
The HMG-box and C1 domains are both essential for binding of CIC to DNA. (A) Diagram of CIC protein constructs tested in EMSA experiments. Constructs 1–3 and 6–17 were transcribed and translated in vitro; constructs 4 and 5 were expressed and purified from bacteria. Construct 2 contains the HMG-box and C1 domains in close proximity, without the intervening sequences that normally separate both domains (see S4 Fig showing that this arrangement is functional in vivo). Construct 10 represents a minimal (min) version of construct 6 where the HMG-box and C1 domains have been placed immediately next to each other. Dashes in the partial sequences of constructs 15 and 16 indicate deleted residues. (B) EMSA analyses of CIC constructs binding to different wild-type and mutant DNA probes. Numbers indicate the constructs used in the binding reactions; unlabeled lanes contain unprogrammed reticulocyte lysate as a negative control. The probes used are indicated below the gels; *1xCBS* and *2xCBS* indicate the presence of 1 or 2 endogenous CIC octameric sites, respectively. *hkb 2xCBS mut* carries mutated CIC sites. The arrowhead marks the position of free, unbound probe in all the gels. Asterisks indicate the differential mobility of protein:DNA complexes. The sequences of wild-type and mutant probes are shown in S1 Table.

Having established that C1 mediates CIC association with endogenous targets, we hypothesized that it might contribute directly to DNA binding. To test this idea, we performed EMSA experiments comparing the binding activities of various *Drosophila* and human CIC constructs. As shown in Fig 5A, these constructs carried intact or mutated HMG-box and C1 domains either alone or combined together in the same polypeptide. These proteins were expressed in vitro or in bacteria, and incubated with probes derived from *Drosophila* and human CIC targets. Unexpectedly, the *Drosophila* HMG-box alone (construct 1) was unable to bind DNA, whereas an HMG-C1 construct containing both domains next to each other (construct 2) showed clear, specific binding to a probe from the *hkb* gene containing two CBSs [12]. Similarly, neither the HMG-box nor the C1 domains alone (constructs 1 and 3) bound to a probe from *pointed* (*pnt*) [14], nor did they bind this probe when combined in the same reaction (Fig 5A and 5B). In contrast, this probe was readily bound by a His-tagged HMG-C1 construct purified from bacteria, but not by the equivalent construct bearing the *cic*^*4*^ lesion (constructs 4 and 5). Likewise, a human HMG-C1 construct efficiently bound to a probe from the *ETV4* gene [15,17], whereas recurrent OD mutations mapping to the HMG-box (R201W and R215W) or C1 (R1515L) domains greatly reduced this binding (constructs 6–9). We also used human HMG-C1 constructs to test the effects of flexible versus rigid linkers separating the HMG-box and C1 domains (constructs 10–12; see Fig 5 legend); both linkers permitted effective binding, ruling out a major effect of the sequences connecting the HMG-box and C1 elements. In contrast, placing the HMG-box and C1 domains in reverse order (construct 13) abolished DNA binding (Fig 5B), indicating that this configuration imposes steric constraints on binding. Thus, the HMG-box and C1 domains function together as an obligate, conformationally oriented module for site-specific binding to DNA.

As indicated above, the C1 sequence contains a highly conserved core of 11 residues flanked by a somewhat more variable amino-terminal extension. To further test the requirements of these sequences in DNA binding, we assayed three mutations of discrete sub-motifs within C1 (constructs 15–17). All three mutations prevented binding of *Drosophila* HMG-C1 to the *hkb* probe (Fig 5B), indicating that these sub-motifs (or a full, correctly folded C1 domain) are important for function.

Finally, we asked if, by analogy to other Sox factors [47–49], the HMG-C1 module binds DNA as a homodimer. To this end, we assayed the binding activities of two HMG-C1 constructs of different size (constructs 2 and 14) using the *pnt* probe, which contains a single CBS. As expected from their relative molecular masses (approximately 24 and 53 kD, respectively), each of these constructs individually produced protein-DNA complexes of different mobility. Similarly, a binding reaction containing both proteins resulted in the same complexes and no intermediate complex was observed (Fig 5B), indicating that the proteins did not oligomerize. These results strongly suggest that C1 does not mediate dimerization and the HMG-C1 module binds DNA as a monomer, although we cannot formally exclude that other CIC sequences may facilitate oligomerization during DNA binding in vivo.

### The HMG-C1 module recognizes discrete octameric sites during DNA binding

**Fig 6:**
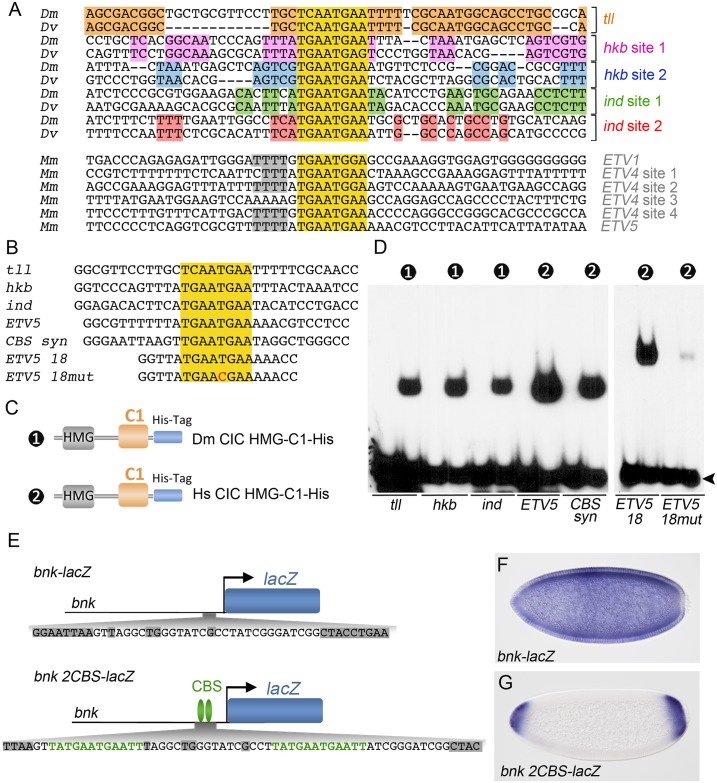
CIC recognizes individual octameric sites and does not depend on helper sites for selecting its targets. (A) Alignment of sequences flanking functional CBSs from selected *D*. *melanogaster* (*Dm*), *D*. *virilis* (*Dv*) and mouse (*Mm*) CIC target genes. The CBSs are highlighted in yellow. Conserved flanking motifs are shaded in different colors. (B) Sequences of probes containing intact or mutated CBSs. (C) Diagram of recombinant *Drosophila* (Dm) and human (Hs) CIC constructs used in the EMSA experiments; both constructs were produced in bacteria. (D) EMSA analyses using the DNA probes and proteins shown in panels B and C, respectively. Numbers indicate the constructs used in the binding reactions; unlabeled lanes are negative controls without added protein. Free probes are indicated by an arrowhead. (E) Diagram of a control *bnk-lacZ* reporter and a modified version carrying two CBSs (*bnk 2CBS-lacZ*). The positions of the inserted CBSs are indicated below the reporters, with conserved motifs among *Drosophila* species shaded in grey. (F, G) Patterns of expression of *bnk-lacZ* and *bnk 2CBS-lacZ* reporters.

The role of C1 in DNA binding is reminiscent of the mechanism employed by certain TCF factors in DNA recognition. Thus, the TCF orthologs from *Drosophila* and *C*. *elegans*, and some vertebrate TCF isoforms, contain, in addition to the HMG-box, a zinc binding domain known as C-clamp which functions in DNA binding. The C-clamp acts by binding so-called ‘Helper sites’ (5’-RCCGCCR-3’) located at short distance (usually <10 bp away) from the sequence recognized by the TCF HMG-box, thereby augmenting the DNA binding strength and specificity of TCF towards its targets [50–56]. Therefore, although the C1 and C-clamp domains are not related in sequence, we considered the possibility that C1 might also recognize a specific conserved motif adjacent to the consensus CBSs. To this end, we first compared the sequences flanking bona fide CBSs present in three *D*. *melanogaster* genes, their *D*. *virilis* orthologs, and three mouse promoters. As shown in Fig 6A, this analysis reveals several conserved motifs in the vicinity of CIC octamers from orthologous *Drosophila* genes, but not across non-orthologous genes. This suggests that those motifs correspond to orthologous sites for other transcription factors in the selected enhancers or promoters. Similarly, the mouse CBSs are flanked by a short A/T-rich extension, but this motif is not well conserved in the *Drosophila* sequences. This indicates that CIC sites are not surrounded by a particular motif serving as a ‘helper site’ for CIC DNA binding.

To directly test the influence in DNA binding of sequences flanking functional CIC octamers, we performed EMSA experiments using probes corresponding to CIC sites present in the *Drosophila tll*, *hkb* and *intermediate neuroblasts defective* (*ind*) genes, and in human *ETV5*. These probes span 30–32 bp and do not share significant similarity outside the CIC octamers (Fig 6B). Nevertheless, they were similarly bound by the corresponding *Drosophila* and human HMG-C1 minimal proteins, indicating that the CIC octamer is the main determinant for DNA recognition in this assay (Fig 6C and 6D). The human protein also bound efficiently a synthetic probe containing a CIC octamer flanked by random sequences (*CBS syn*). Finally, we tested the binding of human HMG-C1 to an 18-bp probe carrying a CBS derived from *ETV5* flanked by only 5 bp on either side (Fig 6B). This probe was bound with similar affinity to that observed using longer probes, and the binding was reduced by a mutation in the CBS (Fig 6D), indicating that a single, isolated CIC octamer is sufficient for effective binding of CIC to DNA.

Finally, we have re-examined if CBSs are sufficient for DNA recognition by CIC in vivo. We selected a 12-bp motif containing a CBS from the *hkb* enhancer and inserted two copies of this sequence in a reporter construct driven by the *bottleneck* (*bnk*) promoter, which is ubiquitously active in early embryos. These insertions were introduced without disrupting conserved elements in the *bnk* promoter, thus preserving its regulation by the Zelda activator and other factors (Fig 6E) [57]. As shown in Fig 6F and 6G, whereas a control *bnk-lacZ* reporter directs uniform expression in the early embryo, the reporter containing CBSs is expressed only in polar regions, indicating that it is effectively regulated by endogenous CIC. This result supports our conclusion that CIC binds its target sites without any requirement or modulation by specific flanking sequences.

### C1-dependent activity of a CIC-DUX4 chimera in *Drosophila*

**Fig 7:**
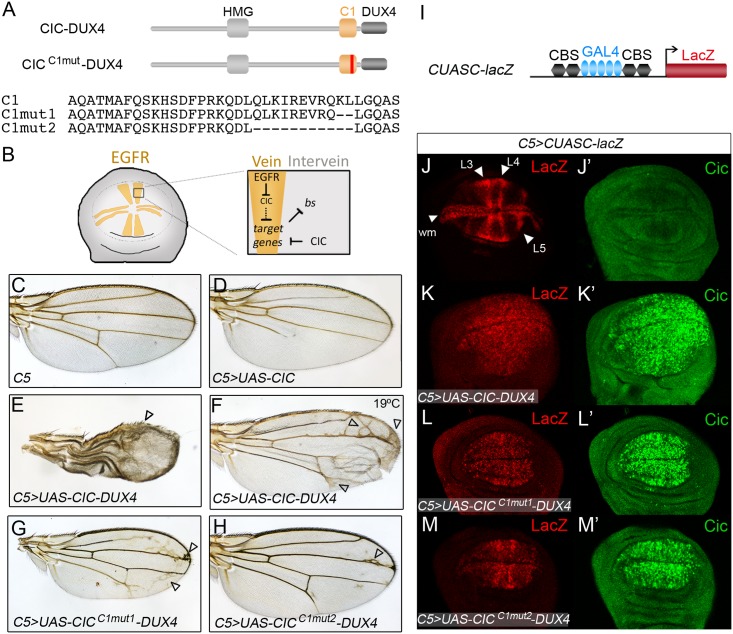
The C1 domain is required for the activity of a CIC-DUX4 fusion in the *Drosophila* wing. (A) Diagram of CIC-DUX4 chimeras expressed in the wing using the GAL4-UAS system. CIC^C1mut^-DUX4 indicates two different derivatives carrying CRISPR-Cas9-induced mutations (vertical red line) in the C1 domain; partial sequences of intact and mutant C1 domains are shown below. (B) Model of CIC function in the wing primordium. The pattern of wing veins is established in the imaginal disc through localized activation of the EGFR signaling pathway, which downregulates CIC in presumptive vein cells (yellow). CIC in turn promotes the intervein fate by repressing (directly or indirectly) EGFR-induced genes such as *ventral veinless* and *decapentaplegic*, while indirectly maintaining *blistered* (*bs*) expression in intervein cells [7,12,67]. (C-H) Wing phenotypes induced by expression of CIC (D), CIC-DUX4 (E, F) and two CIC-DUX4 mutant derivatives carrying deletions in the C1 domain (G, H) under the control of the *C5* GAL4 driver; a control wing with GAL4 driver only is shown in C. Arrowheads indicate broadened veins and ectopic vein material in CIC-DUX4-expressing wings. Unless otherwise indicated, all panels were obtained by raising flies at 25°C; panel F shows the weaker phenotype resulting from induction of CIC-DUX4 at 19°C. (I) Diagram of the *CUASC-lacZ* reporter driven by a synthetic enhancer composed of five GAL4 binding sites flanked by two CBSs on either side. (J-M’) Late third-instar wing discs doubly stained with anti-lacZ (J-M) and anti-Cic antibodies (J’-M’). J and J’ show a wild-type disc carrying the *CUASC-lacZ* reporter. K-M’ show representative discs expressing intact and mutant CIC-DUX4 proteins using the *C5* driver, which is expressed in the wing pouch and serves to activate both the effector genes and the *CUASC-lacZ* reporter.

The above results provide a plausible mechanistic explanation for the main pattern of oncogenic CIC-DUX4 chimeras (which usually include the C1 domain, as shown in Fig 1), since C1 should promote CIC-DUX4 activity by enhancing its binding to DNA. Pursuing this idea, we have established a *Drosophila* assay of CIC-DUX4 activity in which to test the requirement of C1. We made a construct encoding *Drosophila* CIC fused to the C-terminal portion of human DUX4 and expressed this chimera in the developing wing (Fig 7A). This tissue is highly sensitive to changes in CIC activity, which normally acts to promote intervein cell fate except in the presumptive veins where it is inhibited by EGFR signaling (Fig 7B). Thus, loss of CIC function produces extra vein material (Fig 2N; S2 Fig), whereas overexpression of CIC suppresses vein formation (Fig 7D).

We find that targeted expression of CIC-DUX4 in the primordial wing blade (see Materials and methods) causes severe defects including reduced wing size, ectopic venation and blistered wings due to loss of adhesion between the two wing surfaces (Fig 7E and 7F). This phenotype is markedly different to that caused by overexpression of intact CIC and actually resembles the loss of CIC function (see S2 Fig), consistent with CIC-DUX4 mediating transcriptional activation instead of repression. To test this further, we assessed CIC-DUX4 activity in the wing imaginal disc using a synthetic reporter, *CUASC-lacZ*, containing CBSs linked to GAL4 binding sites (Fig 7I). In discs expressing GAL4 protein in the wing pouch, the reporter is activated only in presumptive vein stripes since it is repressed by CIC in intervein regions (Fig 7J) [12]. In contrast, this pattern appears markedly broadened in CIC-DUX4-expressing discs (Fig 7K), as expected if CIC-DUX4 activates the reporter and overrides the repressor activity of endogenous CIC. We then evaluated the contribution of the C1 domain to CIC-DUX4 activity in this assay. Using CRISPR-Cas9, we edited the CIC-DUX4-expressing transgene and isolated two mutations deleting either 2 or 11 residues within the C1 domain of CIC-DUX4 (Fig 7A). Both mutations strongly suppressed the phenotypes produced by CIC-DUX4, with the 11-residue deletion showing almost complete restoration of the wild-type vein pattern (Fig 7G and 7H). This mutant also showed significantly restricted expression of the *CUASC-lacZ* reporter (Fig 7M). Thus, the C1 domain is required for the opposing activities of CIC and CIC-DUX4 proteins in the *Drosophila* wing, which is consistent with its role in DNA binding rather than transcriptional repression per se.

## Discussion

**Fig 8:**
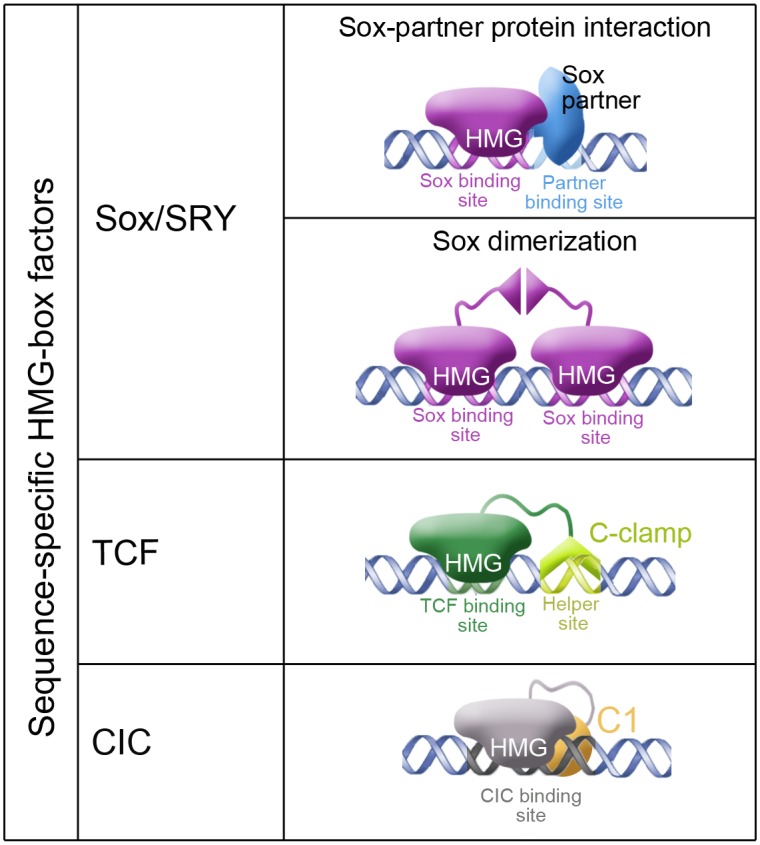
Distinct modes of target recognition by sequence-specific HMG-box proteins. The diagram summarizes the main DNA-binding mechanisms used by each HMG-box sub-family. Sox proteins usually bind their Sox sites in combination with partner factors that recognize adjacent DNA sequences, but can also form homo- and heterodimers via specific dimerization motifs such as those present in SoxD and SoxE family members. Some TCF factors also exhibit bi-partite DNA recognition via the HMG-box and the C-clamp domain that binds GC-rich sequences known as Helper sites. In contrast, CIC proteins appear to bind individual octameric sites through their HMG-box and C1 domains, acting independently of other specific DNA sites and partner proteins.

HMG-box proteins play critical roles in development and disease by regulating the expression of specific target genes. For both Sox and TCF factors, this control depends on the HMG-box as well as on other DNA-binding and dimerization motifs that cooperate in regulating the correct genomic targets. For instance, Sox proteins typically associate with partner factors that interact with specific DNA sequences close to the Sox sites. Similarly, several TCF isoforms contain a C-clamp domain that recognizes GC-rich motifs adjacent to TCF sites, thereby enhancing the affinity and specificity of TCF binding to its targets. It is believed that such combinatorial modes of DNA recognition are essential for proper developmental regulation by both protein families (see Fig 8).

In this work, we have identified a distinct mode of DNA binding by CIC, which depends on its conserved C1 domain. Compared to the above examples, the C1 motif is unique in that it is located at long distance from the HMG-box, does not display detectable DNA-binding activity on its own, does not mediate dimerization, and is not involved in recognizing auxiliary motifs next to CIC octameric sites. Instead, our results indicate that C1 cooperates with the HMG-box to recognize discrete octameric sites both in vitro and in vivo. Since mutations in the C1 domain do not completely abolish the activity of CIC in flies or in human cells (S2 Fig; Fig 4), we favor the view that C1 acts by potentiating the binding of the HMG-box to its specific sites. Several mechanistic models could account for C1 function. For example, C1, like many DNA binding sequences, contains several conserved basic residues in its core, which might establish direct, low-affinity contacts with DNA. Alternatively, the C1 domain could interact with the HMG-box or modulate its folding during DNA recognition. Future high-resolution structural analyses of the HMG-box-C1 module bound to DNA should elucidate the molecular basis of C1 function.

Regardless of the precise molecular mechanism, our results reveal a unique mode of DNA binding that distinguishes CIC from Sox and TCF factors (Fig 8). Thus, the HMG-box-C1 module mediates robust and specific binding to its conserved octameric sites independently of partner factors and auxiliary target sequences. Indeed, CIC recognizes their octameric sites even when those sites are relocated to heterologous or synthetic enhancers (Fig 6G; see also refs. [11,12,19,31]), and our current work demonstrates efficient binding of the HMG-box-C1 polypeptide to an isolated CIC octamer in vitro. It thus appears that HMG-box proteins share a general principle of augmenting their target specificity through modular or cooperative DNA binding, but each individual HMG-box family relies on unique domains and mechanisms for this activity. Furthermore, the distinct binding modes of Sox and CIC proteins give rise to different logics of transcriptional control. Thus, the ‘partner mechanism’ of Sox proteins is highly versatile and leads to either transcriptional activation or repression depending on the partner protein as well as on the promoter context. In contrast, in all cases studied so far, CIC proteins function as dedicated repressors, and *Drosophila* CIC has been shown to contain an intrinsic repressor motif [37].

Finally, our results imply that the two main subgroups of CIC amino acid substitutions in OD and other tumors, which map to the HMG-box and C1 domains (Fig 1A), cause related defects in DNA binding. This would then lead to derepression of CIC targets such as *ETV/PEA3* genes, which encode ETS transcription factors extensively implicated in tumorigenesis, as well as genes encoding feedback inhibitors of RTK signaling like Sprouty and Spred [23,29,30]. Moreover, our findings help explain the main pattern of oncogenic translocations resulting in CIC-DUX4 sarcomas (Fig 1B): it is not incidental that C1 is preserved in most CIC-DUX4 chimeras, since C1 should be required for effective CIC-DUX4 DNA binding and subsequent aberrant activation of *ETV* genes and other targets. This is supported by our analyses (Fig 7) showing that an intact C1 domain is required for the activity of a CIC-DUX4 chimera in the *Drosophila* wing.

## Materials and methods

### *Drosophila* genetics and transgenic lines

The *cic*^*4*^ allele was generated by CRISPR-Cas9-mediated editing. Briefly, a custom gRNA expression construct targeting the C1 coding sequence was prepared in vector *pCDF3* [58] and inserted at the *attP40* landing site via phiC31-mediated integration [59] (see S1 Fig for details of the gRNA sequence). Transgenic gRNA males were crossed to *nanos-cas9* females to obtain founder males, which were then crossed to females carrying the *TM3* balancer for recovery of mutant alleles. Induced mutations were characterized by sequencing PCR fragments amplified from candidate flies. A similar scheme using the same gRNA insertion was employed to isolate mutations in the *UAS-CIC-DUX4* transgene. Other alleles and chromosomal rearrangements employed were: *cic*^*Q474X*^ [10], *cic*^*1*^ [5], *Df(3R)ED6027* (see FlyBase), and the *mirr*^*P2*^ enhancer trap (*mirr-lacZ*; ref. [60]). Transgenic flies expressing CIC derivatives were obtained by P-element transformation. Expression of CIC-DUX4 derivatives was achieved using the GAL4-UAS system and the driver line *C5* [61]. All crosses were performed at 25°C, unless otherwise noted.

### Histochemistry

Embryos were fixed in 4% formaldehyde-PBS-heptane using standard procedures. Ovaries and wing discs were dissected in PBS and fixed with 4% formaldehyde-PBS. In situ hybridizations were performed using digoxigenin-UTP (*kni*, *twi* and *Sxl*) or biotin-UTP (*tll*) labeled antisense RNA probes, followed by incubation with fluorochrome-conjugated anti-digoxigenin or anti-biotin antibodies for FISH analysis, or with secondary antibodies coupled to alkaline phosphatase (AP) for histochemical detection. *Drosophila* CIC was detected using either a guinea pig polyclonal antibody raised against the C-terminal region of the protein [14], or a rabbit polyclonal recognizing the HMG-box and C-terminal regions. Lac-Z and HA-tagged proteins were detected using monoclonal antibodies 40-1a (Developmental Studies Hybridoma Bank) and 12CA5 (Roche), respectively. Immunofluorescence signals were visualized with species-specific secondary antibodies labeled with different fluorochromes (Molecular Probes). Fluorescent and AP-stained samples were mounted in Fluoramount and Permount, respectively. Cuticle preparations were mounted in 1:1 Hoyer’s medium/lactic acid and cleared overnight at 60°C. Wings were rinsed in isopropanol and mounted in Euparal.

### Constructs

The reference sequences used for the *Drosophila* and human CIC proteins are NP_524992.1 and NP_055940.3, respectively. The *CIC(bHLH)* and *CIC(bHLH)*^Δ*C1*^ constructs were made using a genomic *cic-HA* rescue transgene in the *pCaSpeR4* vector [9,62], by replacing an EagI fragment encoding amino acids 384–583 of CIC (including the HMG-box) with a fragment encoding residues 25–150 of Hairy (containing the bHLH domain). *CIC(bHLH)*^Δ*C1*^ carries, in addition, a deletion of the region coding for the C1 domain (residues 1308–1356). The *CIC-DUX4* transgene encodes most of *Drosophila* CIC protein (residues 1–1380) fused to amino acids 325–424 of DUX4 (thus mirroring the chimera described in ref. 31), and was assembled in *pUAST*.

The constructs used in the EMSA experiments express the following CIC amino-acid fragments: 478–572 (Dm CIC HMG), 478–572 fused to 1288–1378 (Dm CIC HMG-C1), 1288–1378 (Dm CIC C1), 188–288 fused to 1451–1527 (Hs CIC HMG-C1), 188–280 fused to 1457–1527 (Hs CIC (HMG-C1)min), 1457–1527 fused to 188–280 (Hs CIC C1-HMG), and 475–598 fused to 1044–1378 (Dm CICmini-DNt). Hs CIC HMG^R201W^-C1, Hs CIC HMG^R215W^-C1 and Hs CIC HMG-C1^R1515L^ are mutant derivatives of Hs CIC HMG-C1. Hs CIC HMG-Flex-C1 and Hs CIC HMG-Rig-C1 are identical to Hs CIC (HMG-C1)min except in that they contain flexible (Flex) and rigid (Rig) linkers separating the HMG-box and C1 domains [63,64]. Dm CIC HMG-C1^mut1-3^ are derivatives of Dm CIC HMG-C1. All these constructs were subcloned into *pET-17b* for in vitro expression under the control of the T7 promoter. His-tagged constructs were expressed in bacteria using the *pET-29b* vector. Dm CIC HMG-C1-His and Dm CIC HMG-C1^ΔRQKL^-His are derivatives of Dm CIC HMG-C1; Hs CIC HMG-C1-His is based on Hs CIC HMG-C1.

GFP-tagged human CIC constructs were assembled in *pcDNA5/FRT/TO* [15]. The R215W and R1515L mutations were introduced using the QuikChange site directed mutagenesis kit (Agilent) following the manufacturer's guidelines. The C1 deletion (spanning residues 1464–1519) was generated using a recombinant PCR-based approach. Unless indicated otherwise, all plasmids were stably introduced into Flp-In T-REx 293 cells (Invitrogen) following instructions from the manufacturer.

### Protein analyses and immunostaining of human cells

For Western blot analysis, cells were lysed in a buffer containing 75 mM NaCl, 50 mM Tris-HCl, pH 8, and 0.5% Triton X-100, supplemented with PMSF and protein inhibitor cocktail Complete Mini (Roche). 50 μg of total protein extract was resolved by SDS-PAGE, transferred to nitrocellulose membranes and probed with antibodies against GFP (Abcam, ab290) and GAPDH (Sigma Aldrich, G8795). To analyze nuclear or cytoplasmic localization of the different CIC constructs, we transiently transfected a plasmid encoding GFP (*pEFGP-C2*) as a control or plasmids encoding WT [15] or mutated GFP-CIC constructs into 293T cells. 48h after transfection, cells were fixed with 4% formaldehyde and permeabilized with 0.5% Triton X-100. GFP expression was detected using polyclonal anti-GFP antibodies (Abcam ab290, 1:1000) followed by counterstaining with Hoechst 33342. Images were acquired with a Leica TCS SP5 confocal microscope.

### Luciferase assay

Luciferase assays were performed in a Glomax luminometer (Promega) according to the manufacturer's guidelines. Briefly, we transfected the *pGL3proERM-338/-329* tandem reporter vector [31] along with empty *pcDNA5/FRT/TO* vector or *pcDNA5/FRT/TO* plasmids expressing wild-type or mutant GFP-tagged human CIC derivatives into 293T cells using jetPRIME reagent (Polyplus-transfection). Cells were lysed after 48 h and assayed for luciferase activity. A Renilla luciferase-expressing vector was used for normalization.

### ChIP assay

ChIP assays were performed as described [65]. Briefly, 2x10^7^ Flp-In T-REx 293 cells stably transfected with *pcDNA5/FRT/TO* alone or *pcDNA5/FRT/TO* expressing either wild-type or mutated (R215W, R1515L or C1 domain deletion) GFP-tagged human CIC cDNAs were cross-linked for 15 min at room temperature. After washing, cells were sonicated at high intensity during 30 cycles, with 30 s ON and 30 s OFF per cycle (Bioruptor Plus, Diagenode), followed by centrifugation for 15 min at 14,000 rpm at 15°C. For each condition, 200 μg of lysate was incubated overnight with 2 μl of anti-GFP antibody (Abcam, ab290) and immunoprecipitated by incubation with 20 μl of protein A/G beads during 1 h at 4°C in a rotating platform. After reverse crosslinking, DNA fragments were recovered by phenol/chloroform extraction and qRT-PCR was carried out in a 7500 Fast Real-Time PCR System (Applied Biosystems) using Power SYBR green PCR Mastermix (Applied Biosystems) with the following primers: *ETV1* promoter, 5-caaccacgtgaccaagaag-3 and 5-GCGCTCCGCTAGGAGATT-3; *ETV4* promoter, 5-cttctctctttttctctcggttc-3 and 5-CCAATCAGAATGTAGGGGTTG-3; *ETV5* promoter, 5-aagtgcttcactgactcagctaa-3 and 5-CATTGGCCAATCAGCACA-3. As a negative control we used a region of the *CDK1* promoter without known CBSs, amplified with primers 5-ggccttcaacgtatgaattagc-3 and 5-AGTTGGTATTGCACATAAGTCT-3.

### In vitro DNA binding assays

EMSA experiments were performed using CIC protein fragments synthesized with the TNT T7 Quick Coupled Transcription/Translation system (Promega). For expression of His-tagged proteins, bacterial cultures were induced for 2 h with 1 mM IPTG and proteins purified using the Proteus IMAC Mini Sample kit. DNA probes were synthesized as complementary oligonucleotides leaving 5’ GG overhangs, or amplified by PCR with primers carrying NotI restriction sites, subcloned, and released by NotI digestion. Probes were then end-labeled using α-^32^P-dCTP and Klenow Fragment, exo- (Thermo Scientific). The sequences of wild-type and mutant probes are shown in S1 Table.

Binding reactions were carried out in a total volume of 20 μl containing 60 mM Hepes pH 7.9, 20 mM Tris-HCl pH 7.9, 300 mM KCl, 5 mM EDTA, 5 mM DTT, 12% glycerol, 1 μg poly(dI-dC), 1 μg BSA, ~1 ng of DNA probe, and 1 μl of programmed or non-programmed (control) TNT lysate (or ~1 ng of bacterially expressed His-tagged protein). After incubation for 20 min on ice, protein-DNA complexes were separated on 5% non-denaturing polyacrylamide gels run in 0.5X TBE at 4°C, and detected by autoradiography.

## Supporting information

S1 Fig Isolation of a CRISPR-Cas9-induced mutation in the C1 motif of CIC. Shown is a diagram of the targeted sequence indicating the protospacer and protospacer adjacent motif (PAM) elements. The predicted cleavage site of Cas9 is indicated by an arrowhead. A sequencing chromatogram of a PCR product amplified from a *cic*^*4*^ homozygous fly is shown below; note the loss of the sequence encoding the RQKL motif.
(TIF)

S2 Fig The *cic*^*4*^ allele is a strong hypomorph. (A-C) Cuticles of embryos derived from females of the indicated genotypes. The *cic*^*1*^ allele is a strong hypomorphic mutation specifically affecting CIC function in the early embryo. *cic*^*Q474X*^ is a nonsense mutation upstream of the HMG-box coding region and behaves as a genetic null. *Df(3R)ED6027* is a deletion that removes the *cic* locus. Embryos from *cic*^*4*^*/cic*^*1*^ females often exhibit small patches of cuticle with ventral denticles (arrowhead in A), indicating some residual differentiation of abdominal structures; in contrast, such denticles are never seen in embryos from *cic*^*Q474X*^*/cic*^*1*^ or *Df(3R)ED6027/cic*^*1*^ females. (D-F) Representative wings from flies of the indicated genotypes. Note that *cic*^*4*^ homozygous mutant wings are less affected (e.g. show less ectopic vein material and blisters) than *cic*^*Q474X*^*/cic*^*4*^ or *Df(3R)ED6027/cic*^*4*^ wings. Thus, *cic*^*4*^ is a weaker allele than *cic*^*Q474X*^ or *Df(3R)ED6027* in the two contexts examined.
(TIF)

S3 Fig Subcellular localization of CIC constructs in human cells. (A-E”) Confocal images of 293T cells transfected with the indicated GFP-tagged constructs and co-stained using anti-GFP antibody (A-E) and Hoechst 33342 (A’-E’). Control expression of GFP alone is shown in A’-A”. Note that all CIC derivatives are localized to the nucleus.
(TIF)

S4 Fig A minimal CIC protein composed of N2, HMG-box and C1 domains is functional in the early embryo. (A) Diagram of the HA-tagged Cic(N2-HMG-C1) derivative. The structural arrangement of the HMG-box and C1 domains is identical to that of construct 2 in Fig 5A. The N2 motif is described in ref. 37. (B) Expression of CIC(N2-HMG-C1)-HA in a blastoderm embryo stained with an anti-HA antibody. The protein was expressed using a transgene under the control of 5’ and 3’ *cic* genomic sequences [9,62]. (C, D) Maternal expression of CIC(N2-HMG-C1) significantly rescues the *cic* mutant (*cic*^*1*^*/cic*^*Q474X*^) phenotype. Note the presence of abdominal denticle belts in the rescued embryo (arrowheads). Panel D shows a control *cic*^*1*^*/cic*^*Q474X*^ cuticle. (E, F) CIC(N2-HMG-C1) rescues the central band of *kni* mRNA expression in *cic*^*1*^*/cic*^*Q474X*^ embryos. A control *cic*^*1*^*/cic*^*Q474X*^ embryo lacking abdominal *kni* expression is shown in F.
(TIFF)

S1 Table Sequences of probes used in EMSA experiments. The table lists the sequences of DNA probes used in Fig 5, with intact and mutated CIC sites highlighted in yellow. References describing the different CIC sites are also indicated.
(TIFF)
