# Supplementary figures and images for: A new mode of DNA binding distinguishes Capicua from other HMG-box factors and explains its mutation patterns in cancer
Source: PLoS Genet. 2017 Mar 9;13(3):e1006622. doi: 10.1371/journal.pgen.1006622 (PMC5344332; doi:10.1371/journal.pgen.1006622)

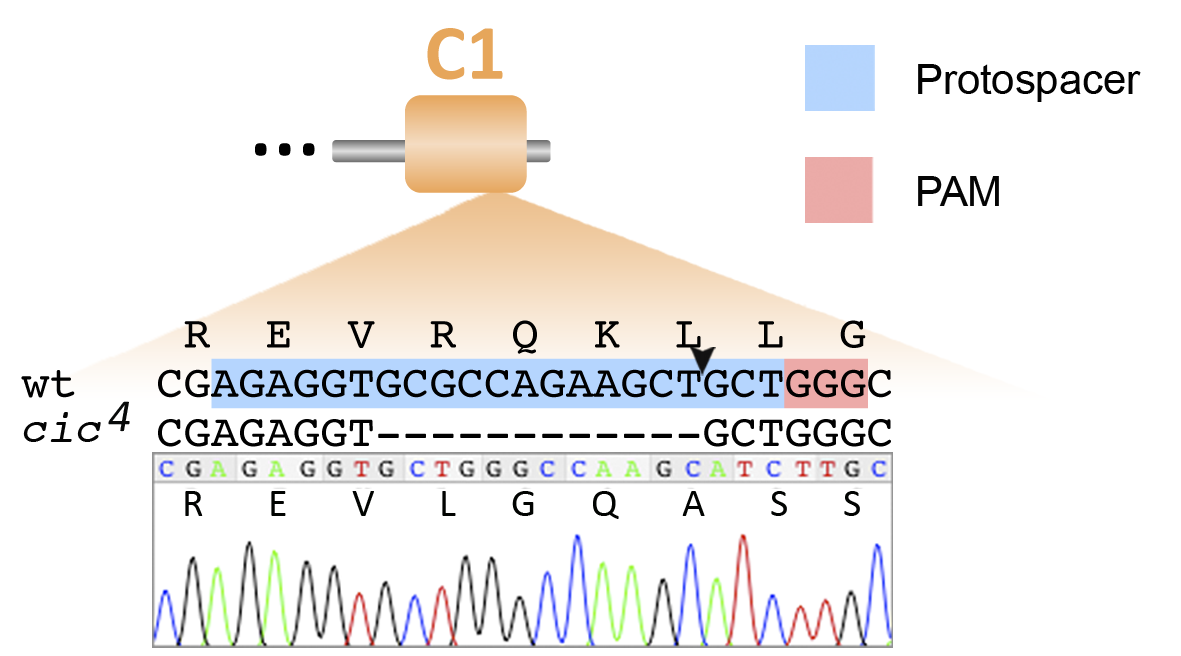

Supplement: S1 Fig — Shown is a diagram of the targeted sequence indicating the protospacer and protospacer adjacent motif (PAM) elements. The predicted cleavage site of Cas9 is indicated by an arrowhead. A sequencing chromatogram of a PCR product amplified from a cic4 homozygous fly is shown below; note the loss of the sequence encoding the RQKL motif. (TIF) [file pgen.1006622.s001.tif]

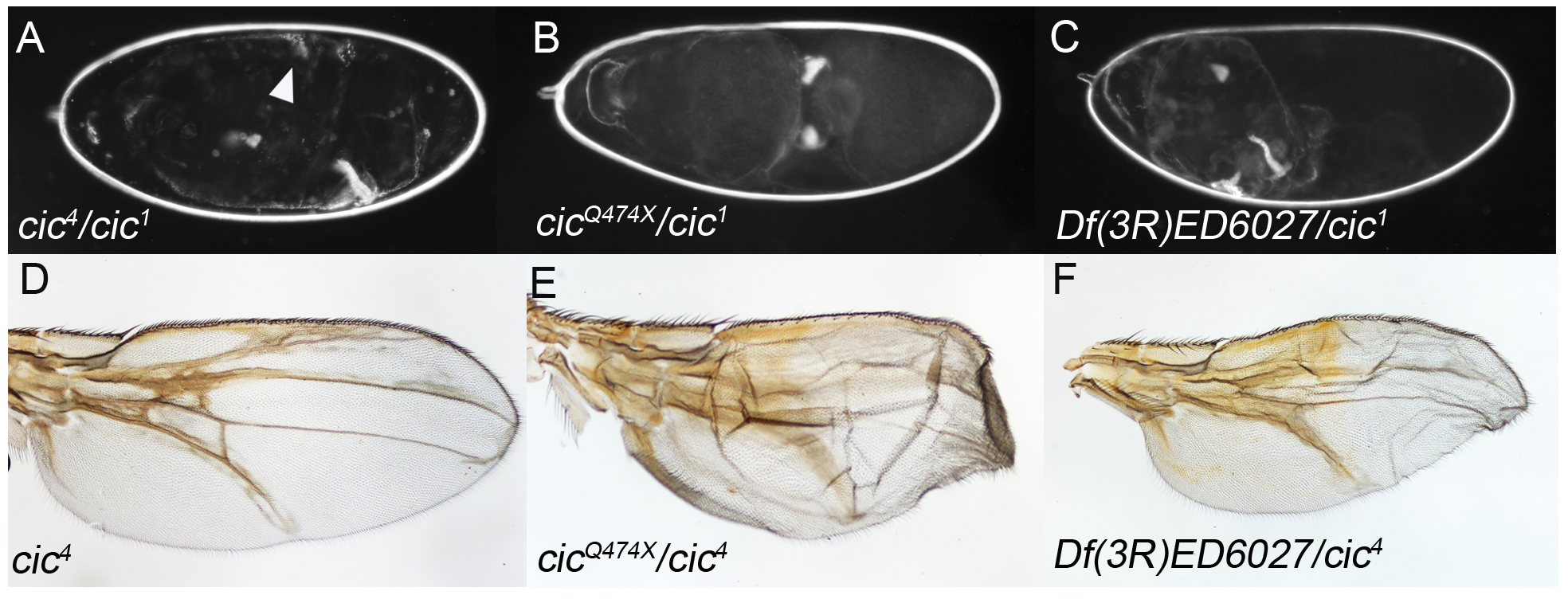

Supplement: S2 Fig — (A-C) Cuticles of embryos derived from females of the indicated genotypes. The cic1 allele is a strong hypomorphic mutation specifically affecting CIC function in the early embryo. cicQ474X is a nonsense mutation upstream of the HMG-box coding region and behaves as a genetic null. Df(3R)ED6027 is a deletion that removes the cic locus. Embryos from cic4/cic1 females often exhibit small patches of cuticle with ventral denticles (arrowhead in A), indicating some residual differentiation of abdominal structures; in contrast, such denticles are never seen in embryos from cicQ474X/cic1 or Df(3R)ED6027/cic1 females. (D-F) Representative wings from flies of the indicated genotypes. Note that cic4 homozygous mutant wings are less affected (e.g. show less ectopic vein material and blisters) than cicQ474X/cic4 or Df(3R)ED6027/cic4 wings. Thus, cic4 is a weaker allele than cicQ474X or Df(3R)ED6027 in the two contexts examined. (TIF) [file pgen.1006622.s002.tif]

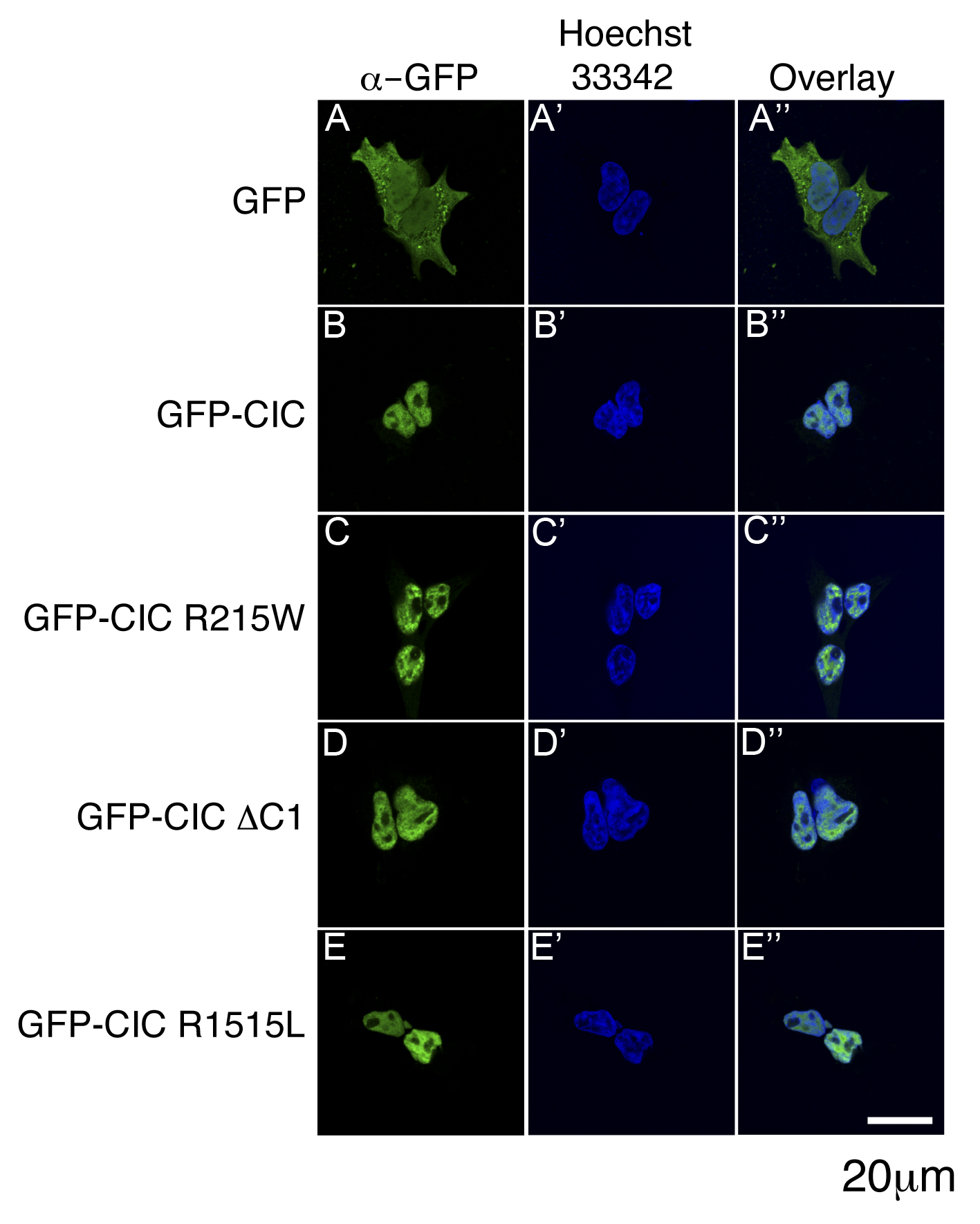

Supplement: S3 Fig — (A-E”) Confocal images of 293T cells transfected with the indicated GFP-tagged constructs and co-stained using anti-GFP antibody (A-E) and Hoechst 33342 (A’-E’). Control expression of GFP alone is shown in A’-A”. Note that all CIC derivatives are localized to the nucleus. (TIF) [file pgen.1006622.s003.tif]

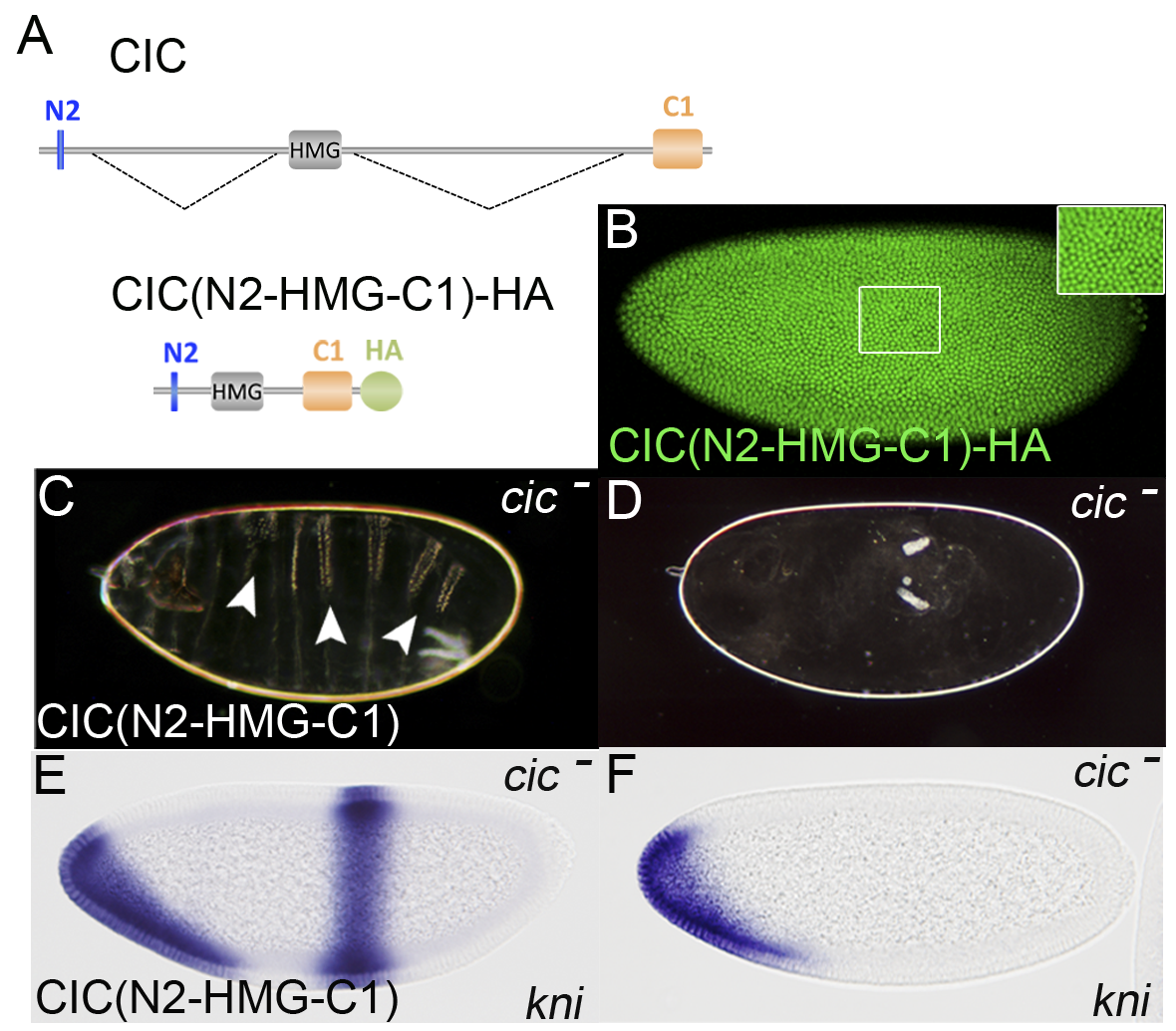

Supplement: S4 Fig — (A) Diagram of the HA-tagged Cic(N2-HMG-C1) derivative. The structural arrangement of the HMG-box and C1 domains is identical to that of construct 2 in Fig 5A. The N2 motif is described in ref. 37. (B) Expression of CIC(N2-HMG-C1)-HA in a blastoderm embryo stained with an anti-HA antibody. The protein was expressed using a transgene under the control of 5’ and 3’ cic genomic sequences [9,62]. (C, D) Maternal expression of CIC(N2-HMG-C1) significantly rescues the cic mutant (cic1/cicQ474X) phenotype. Note the presence of abdominal denticle belts in the rescued embryo (arrowheads). Panel D shows a control cic1/cicQ474X cuticle. (E, F) CIC(N2-HMG-C1) rescues the central band of kni mRNA expression in cic1/cicQ474X embryos. A control cic1/cicQ474X embryo lacking abdominal kni expression is shown in F. (TIFF) [file pgen.1006622.s004.tiff]

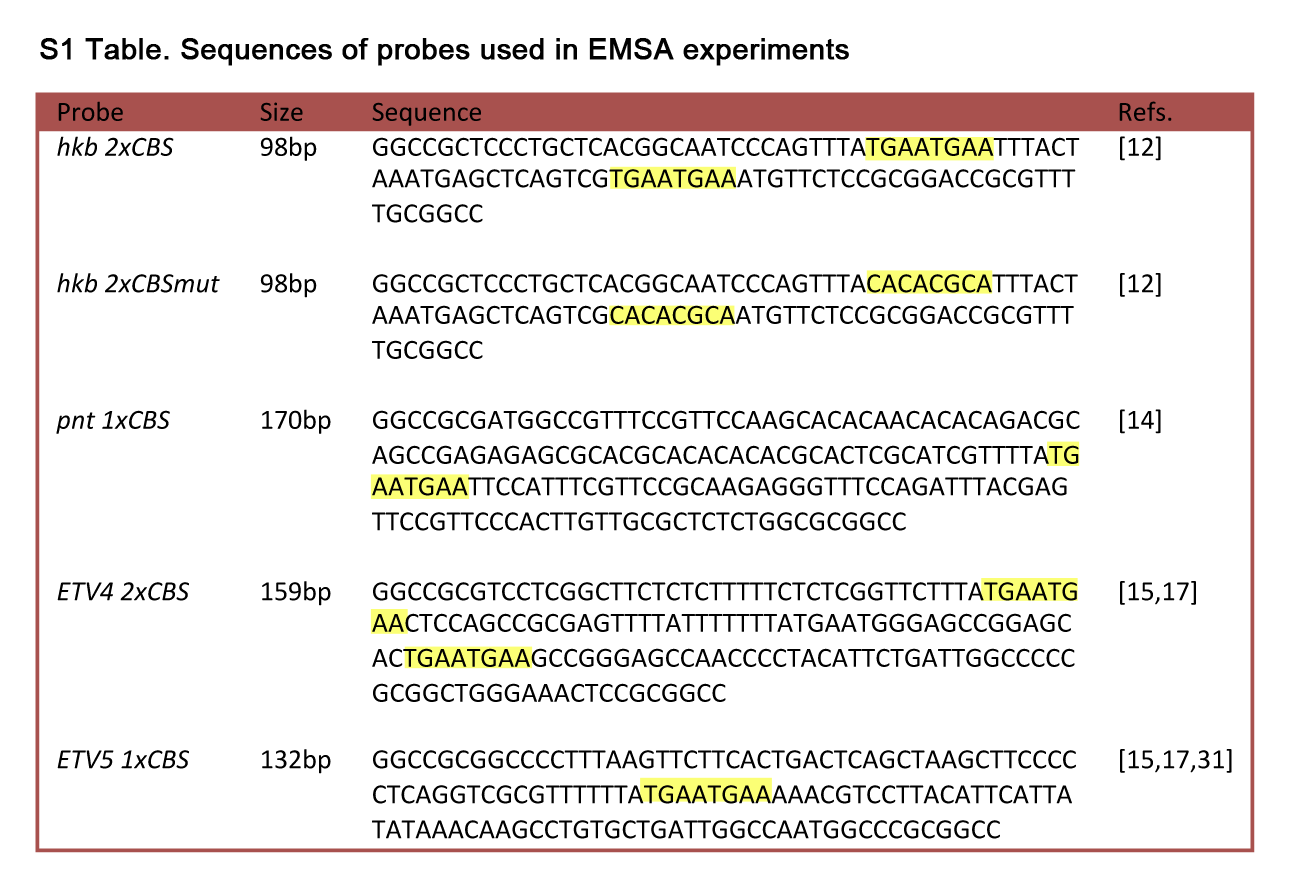

Supplement: S1 Table — The table lists the sequences of DNA probes used in Fig 5, with intact and mutated CIC sites highlighted in yellow. References describing the different CIC sites are also indicated. (TIFF) [file pgen.1006622.s005.tiff]
